# Functional Study of Opsin Genes in *Pardosa astrigera* (Araneae: Lycosidae)

**DOI:** 10.3390/insects16060595

**Published:** 2025-06-05

**Authors:** Shuxin Zhai, Boqi Ren, Xinghua Zhang, Fangyu Shen, Min Ma, Xinmin Li, Rui Li

**Affiliations:** 1 College of Plant Protection, Shanxi Agricultural University, Jinzhong 030801, China; zhaishuxin2000@163.com (S.Z.); renboqi2024@163.com (B.R.); zxhbebetter@163.com (X.Z.); 18730773282@163.com (F.S.); sxndminde@163.com (M.M.); 2 Shanxi Key Laboratory of Integrated Pest Management in Agriculture, Shanxi Agricultural University, Jinzhong 030801, China; 3 Technology Center for Genomics and Bioinformatics, Department of Pathology and Laboratory Medicine, University of California, Los Angeles, CA 90095, USA; xinminli@mednet.ucla.edu

**Keywords:** opsin, biological control, phototactic behavior, *Pardosa astrigera*, vision

## Abstract

Spiders are important predatory natural enemies in agricultural and forestry ecosystems, yet the role of vision in their predatory behavior remains unclear. In this study, we screened three opsin genes—corresponding to ultraviolet-sensitive and medium-to-long wavelength-sensitive opsins—from the transcriptome sequencing database of *Pardosa astrigera*. All three genes possess seven transmembrane topological structures and a lysine residue on the second transmembrane domain, which are typical characteristics of opsins. Using quantitative real-time PCR (RT-qPCR), we analyzed the expression patterns of these opsin genes in different tissues, developmental stages, and under the induction of light at three wavelengths. The results showed that all three opsin genes were significantly expressed in the cephalothorax and expressed across developmental stages with no significant differences. Under light induction, their relative expression first increased and then decreased in both male and female adult spiders. Subsequently, RNA interference (RNAi) was used to individually knock down each opsin gene, confirming their involvement in color vision. These results suggest that the three opsin genes are involved in spider vision, laying the foundation for further elucidating the role of vision in spider predation, and offering a new perspective for reducing the unintended killing of natural enemies by insect traps.

## Simple Summary

The mechanism of vision underlying the survival and predation behavior of *Pardosa astrigera* remains unclear, prompting us to investigate its vision-related genes at the molecular level. In this study, we performed functional validation of *P. astrigera* opsin genes using quantitative real-time PCR (RT-qPCR) and RNA interference (RNAi) techniques. Our results demonstrated that these genes are expressed across various developmental stages and tissues. Moreover, their expression levels increased and then decreased following exposure to different wavelengths of light. Gene silencing experiments revealed that interference with opsin genes leads to the loss of wavelength-specific selectivity. These findings indicate that Opsin genes exhibit high expression levels in the cephalothorax, consistent with their primary role in visual perception. However, their detectable expression in the abdomen and legs—tissues lacking visual function—suggests that these genes may also be involved in non-visual biological processes. We propose that *P. astrigera* possesses trichromatic vision, and its selective color perception may have practical implications for its use in biological control strategies.

## 1. Introduction

As vital natural enemies in agricultural and forestry ecosystems, spiders exhibit many advantageous traits such as agility, wide distribution, high species diversity, large population size, strong predation capabilities, rapid reproduction, high adaptability, and long lifespan. Nowadays, over 52,000 spider species have been recorded [1]. Amid increasing concerns about the 3Rs (resistance, residue, resurgence) in agricultural development, biological control and pest control using natural enemies have gained importance. Spiders play a critical role in agricultural ecosystems. It is estimated that spiders collectively kill 400 to 800 million tons of insect prey globally every year [2]. In both agricultural and non-agricultural habitats, spiders contribute an average of 4.9% to insect predation [3]. For instance, they help reduce pear psyllids in orchards, aphids in apple orchards, and rice planthoppers in paddies [4,5,6]. Some scholars even prove that the abundance of spider populations can be promoted through the adjustment of composite agroforestry ecosystems, plant landscape configurations, and farming systems, and the potential for controlling pests can be improved [7,8,9]. Thus, spiders help stabilize ecosystems through predation, indirectly ensuring agricultural safety and product quality.

Spiders locate and hunt prey using multiple sensory modalities, including olfaction, vision, hearing, touch, and taste. Raška et al. [10] observed that jumping spiders (*Evarcha arcuata*) can detect chemical signals from the volatile compounds of *Pyrrhocoris apterus* larvae and Liu et al. [11] found that even volatile rice induced by herbivores can be used to attract spiders and improve control of rice pests. Barth [12] demonstrated that spider hairs serve as auditory organs, capable of detecting mechanical energy from the environment to help them capture flying insects [13]. Mortimer [14] observed that spiders can distinguish different web vibrations and respond accordingly. Ganske and Uhl [15] identified chemosensory receptors associated with taste via scanning electron microscopy, and Lin et al. [16] noted the involvement of taste in courtship and mating behavior.

Vision is especially important for prey detection, localization, and capture in some spider families like Salticidae (jumping spiders), Lycosidae (wolf spiders), and Thomisidae (crab spiders). Spiders have eight single-lens eyes divided into principal and secondary eyes: the former recognize stationary objects, and the latter detect motion [17]. Using intracellular recordings, De Voe [18] identified three types of photoreceptors in the principal eyes of *Phidippus regius*, suggesting potential dichromatic vision. Barth et al. [19] recorded the spectral sensitivity of *Cupiennius salei* using electroretinograms (ERGs) and found peaks at 520 or 540 nm, a shoulder at 340–380 nm, and another minor peak at 480 nm, suggesting the presence of two to three opsins. Walla et al. [20] confirmed the existence of UV, blue, and green photoreceptors. Koyanagi et al. [21] identified three opsin genes in *Hasarius adansoni* and *Plexippus paykulli*, while Zopf et al. [22] found similar opsins in *C. salei*. One opsin (*RH3*) was UV/blue-sensitive, and *RH1* and *RH2* were medium-to-long-wavelength-sensitive. Nakamura & Yamashita [23] showed that *H. adansoni* could discriminate between colored papers. Huang et al. [24] found that *Pardosa pseudoannulata* was more sensitive to red (625–740 nm) and green (500–565 nm) light, indicating the existence of color vision in spiders.

With the development of integrated pest management (IPM), physical control tools like light traps and sticky cards have been widely adopted due to their low cost, simplicity, and safety. However, because phototactic behavior is common among insects, these tools often lack target specificity and may capture both pests and beneficial insects—up to 46.9% of non-target insects are caught [25]. This disrupts the balance between pests and natural enemies and decreases biodiversity in agroecosystems. Spiders, as crucial natural enemies, are also affected.

**Figure 1:**
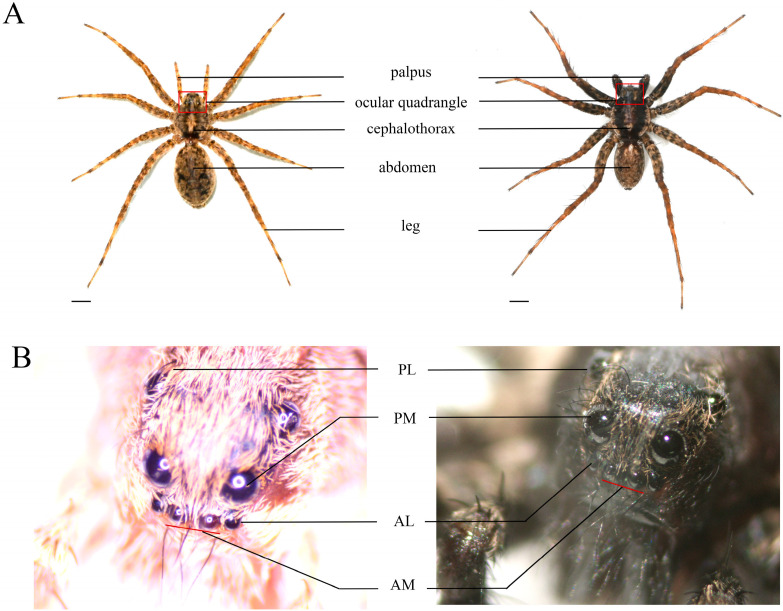
The adult male and female *P. astrigera* and their eye arrangement diagram. (**A**) Female and male; (**B**) Ocular quadrangle of female and male. PL: aposterior lateral, PM: aposterior median, AL: anterior lateral, AM: anterior median. Scale bars = 1 mm.

*Pardosa astrigera*, a wolf spider of the genus *Pardosa* (Figure 1A), is known for its large population size, broad prey spectrum, high predation capacity, strong reproductive ability, long adult lifespan, strong starvation tolerance, and aggressive nature. As a wandering hunter, it actively roams or hunts on the ground, in grasslands, and among plant branches and leaves, with males being particularly agile and active. It is widely distributed in China and is a dominant or common species in various agricultural habitats, with important research and development value. In this study, we used *P. astrigera*, which possesses eight simple eyes (Figure 1B), to investigate the role of opsins in phototactic behavior. We hypothesized that opsin genes are key regulators of wavelength-specific light preference. We evaluated whether down-regulating these genes would affect spiders’ phototactic responses. First, three putative vision-related genes were identified from a transcriptome database (Table S1). Then, RT-qPCR was used to analyze their expression patterns across tissues, developmental stages, and light treatments. Finally, RNA interference (RNAi) and behavioral assays were conducted to identify the key genes affecting phototactic behavior. This study expands our understanding of spider behavior, lays a foundation for communication studies within and between species, and provides theoretical support for using *P. astrigera* in pest biological control.

## 2. Materials and Methods

### 2.1. Spider Collection and Breeding

A laboratory colony of *P. astrigera* was originally established from individuals collected in a wheat field in Shanxi Province, China. They were reared individually in glass tubes (1.5 cm in diameter, 8 cm high) with moistened cotton for humidity. They were kept in an incubator (Percival, Perry, IO, USA) under controlled conditions: 26 ± 1 °C, 60 ± 10% relative humidity, and a 14:10 h light/dark photoperiod. The spiders were fed *Drosophila melanogaster* prior to the third instar and *Tenebrio molitor* thereafter. Unmated male and female adult spiders were paired for mating, and successfully mated females were kept individually for oviposition. Hatched spiderlings were used as experimental subjects in this study.

### 2.2. Identification, Cloning, and Analysis of Opsin Gene

Opsin genes were identified from the existing transcriptome database of *P. astrigera*. Primers were designed using Primer Premier(6.25, Premier Biosoft, Palo Alto, CA, USA) and synthesized by Sangon Biotech (Shanghai, China). Primer information is in Table S2. Total RNA was extracted from male and female adults using the UNIQ-10 Column Total RNA Purification Kit (Sangon, Shanghai, China) and verified by agarose gel electrophoresis and a micro-spectrophotometer. RNA samples were stored at −80 °C. First-strand cDNA was synthesized using HiScript II 1st Strand cDNA Synthesis Kit (Vazyme, Nanjing, China) with Oligo(dT)23VN primers and stored at −20 °C.

PCR was conducted using TransStart^®^ FastPfu Fly DNA Polymerase (TransGen, Beijing, China), with a 50 μL reaction system. PCR products were detected by agarose gel electrophoresis, and target bands were recovered using a DNA Gel Extraction Kit (Sangon, Shanghai, China). Purified DNA fragments were cloned into the pEASY^®^-T&B Zero vector (TransGen, Beijing, China) for transformation. Positive clones were verified by colony PCR and sequenced.

Amino acid sequences were aligned using ClustalW in MEGA (11.0.13, MEGA Team, Philadelphia, PA, USA). Phylogenetic trees were inferred using the Neighbor-Joining method [26]. The percentage of replicate trees in which the associated taxa clustered together in the bootstrap test (1000 replicates) are shown next to the branches [27]. The evolutionary distances were computed using the JTT matrix-based method [28]. All ambiguous positions were removed for each sequence pair. Evolutionary analyses were conducted in MEGA 11 [29] and visualized in iTOL (https://itol.embl.de/) accessed on 23 May 2025. Conserved motifs were predicted with Motif Scan (http://myhits.isb-sib.ch/cgi-bin/motif_scan) accessed on 2 January 2025, transmembrane helices with TMHMM 2.0 (https://services.healthtech.dtu.dk/service.php?TMHMM-2.0) accessed on 2 January 2025, signal peptides with SignalP 4.1 (https://services.healthtech.dtu.dk/service.php?SignalP-4.1) accessed on 2 January 2025, and subcellular localization with WoLF PSORT (https://wolfpsort.hgc.jp/) accessed on 2 January 2025.

### 2.3. Different Tissue, Development Stage and Light-Induced Expression of Opsin Genes

For comparison of RNA expression across different tissues, thirty male and thirty female adults were starved for three days, then dissected on ice after flash-freezing in liquid nitrogen. Tissues collected included the cephalothorax, abdomen, and legs. RNA was extracted and reverse-transcribed to cDNA. RT-qPCR was performed using ChamQ Universal SYBR qPCR Master Mix (Vazyme, Nanjing, China) with *β-actin* as the reference gene. Each reaction (20 μL) included 10 μL of the master mix, 0.4 μL of forward and reverse primers, 2 μL of cDNA, and 7.2 μL of ddH_2_O. Each treatment had three biological replicates and three technical replicates.

For developmental staging assays, one unmated adult male and one unmated adult female *P. astrigera* were gently placed together in a 25 mL Erlenmeyer flask containing a thoroughly moistened cotton ball to maintain adequate humidity, allowing them to successfully mate and oviposit. After hatching from the delicate egg sac, the spiderlings tightly clustered on the female’s cephalothorax and abdomen, clearly exhibiting a striking form of maternal care behavior. Once the spiderlings molted and actively dispersed from the female’s body, they were carefully collected and defined as second-instar juveniles. Thereafter, each distinct molt was considered indicative of progression to the subsequent developmental stage.

For comprehensive RNA extraction, individuals were meticulously collected at multiple developmental stages: 30 s-instar, 25 third-instar, 15 fourth-instar, 5 fifth-instar and sixth-instar individuals, as well as 2 adult males and 2 adult females. Total RNA was precisely extracted from each developmental stage, standardized to a final concentration of 50 ug/uL, reverse-transcribed into cDNA, and rigorously subjected to RT-qPCR analysis. Each experimental treatment included three independent biological replicates and three technical replicates per biological replicate.

For light induction assays, healthy adult male and female *P. astrigera* were first acclimated in darkness for 2 h at 29 ± 1 °C. Subsequently, individuals were exposed to ultraviolet light (370–375 nm), blue light (480–485 nm), or green light (520–525 nm). Exposure durations were set at 0 min (control), 10 min, 20 min, 30 min, 60 min, and 120 min. After each treatment, total RNA was extracted from the spiders, reverse transcribed into cDNA, and subjected to RT-qPCR analysis. Each treatment was performed with three biological replicates, and each replicate consisted of two spiders.

### 2.4. RNAi-Mediated Knockdown of Opsin Gene Expression

To reduce opsin expression, RNA interference (RNAi) was used. Plasmids were extracted using TIANprepare Mini Plasmid Kit (Tiangen, Beijing, China). Gene fragments were PCR-amplified with T7 promoter-linked primers and used as templates for dsRNA synthesis with the T7 RiboMAX^TM^ Express RNAi System (Promega, Madison, WI, USA). dsRNA for *eGFP* served as a negative control. dsRNA integrity and concentration were verified by agarose gel electrophoresis and spectrophotometry.

Each dsRNA was mixed with nanomaterials (Provided by Professor Shen Jie from the School of Plant Protection, China Agricultural University) at a 1:1 mass ratio and incubated for 15 min at room temperature, followed by the addition of 10% surfactant to reduce surface tension. The final dsRNA concentration was 1 μg/μL, and 2 μL was topically applied to each spider.

At 24, 48, and 72 h post-treatment, spiders were collected for RNA extraction and RT-qPCR as described. Each treatment included three biological replicates and three technical replicates.

### 2.5. Behavioral Verification

**Figure 2:**
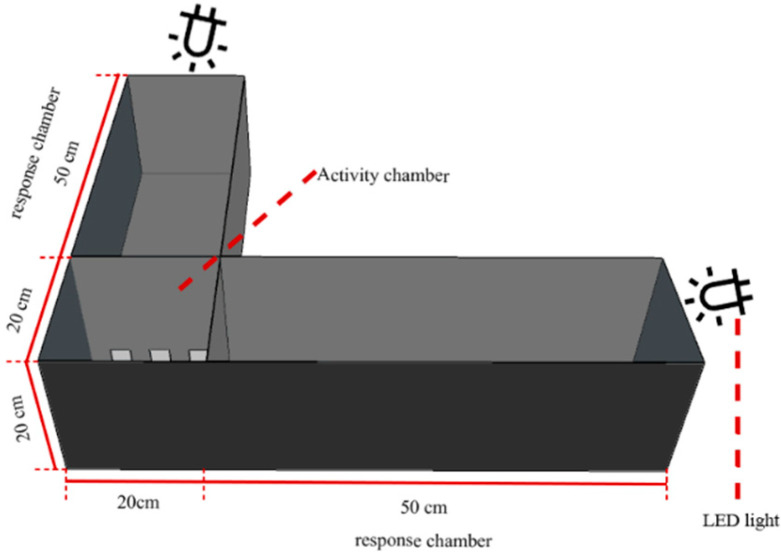
Experimental device of selection response of *P. astrigera* to light source.

A phototactic behavior assay chamber was built based on the research by Zhang et al. [30]. The device included a reaction chamber (50 × 20 × 20 cm) and an activity chamber (20 × 20 × 20 cm), made of 3 mm thick black opaque polypropylene boards. Chambers were connected by a partition with three square openings (3 cm side length). A mesh cover was placed on the activity chamber to prevent escape. The experimental apparatus of female *P. astrigera* responding to light source selection is shown in Figure 2.

RNAi-treated spiders were placed in the activity chamber and observed for 5 min. If a spider entered and remained in any part of the reaction chamber for over 1 min, the response was recorded as valid; otherwise, it was considered invalid. The positions of two LED lightboards was randomly swapped before each test (Video S1). Three replicate cohorts were tested, with each cohort being composed of 10 spiders per experimental condition. Each spider was tested only once, and unresponsive individuals were excluded. GFP-dsRNA-treated spiders served as the control.

### 2.6. Data Analysis

Experimental data were analyzed using SPSS (27.0.1.0, IBM, Amonk, NY, USA) with single factor ANOVA test, independent-sample *t*-tests, and chi-square tests. Relative expression level was calculated using the 2^−ΔΔCt^ method. Graphs were visualized using Origin (10.1, OriginLab, Northampton, MA, USA) software.

## 3. Results

### 3.1. Identification of Opsin-Related Genes

**Figure 3:**
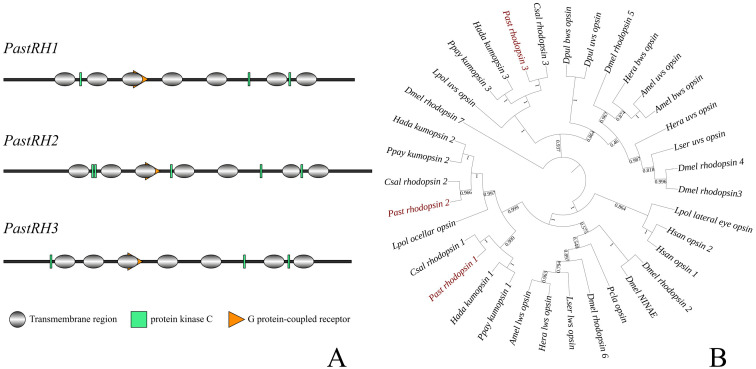
(**A**) Opsin genes conservative structure domain analysis (**B**) Opsin genes phylogenetic analysis (Csal: *Cupiennius salei*: the GenBank accession numbers of *rhodopsin1*, *rhodopsin2*, and *rhodopsin3* are CCO61973.1, CCO61974.1, and CCO61975.1, respectively. Dmel: *Drosophila melanogaster*: the GenBank accession numbers of *NINAE*, *rhodopsin2*, *rhodopsin3*, *rhodopsin4*, *rhodopsin5*, *rhodopsin6*, *rhodopsin7* are NP_524407.1, NP_524398.1, NP_52441.1, NP_476701, NP_477096.1, NP_524368.5, and NP_524035.2, respectively. Amel: *Apis mellifera*: the GenBank accession numbers of *lws opsin*, *bws opsin* and *uvs opsin* are NP_001011639.2, AAC13417.1 and AAC47455.1, respectively. Hada: *Hasarius adansoni*: the GenBank accession numbers of *kumopsin1*, *kumopsin2* and *kumopsin3* are BAG14330.1, BAG14331.1 and BAG14332.1, respectively. Ppay: *Plexippus paykulli*: the GenBank accession numbers of *kumopsin1*, *kumopsin2* and *kumopsin3* are BAG14333.1, BAG14334.1, and BAG14335.1. Past: *Pardosa astrigera*: the GenBank accession numbers of *rhodopsin1*, *rhodopsin2*, and *rhodopsin3* are PV524665.1, PV524666.1, PV524667.1. Dpul: *Daphnia pulex*: the GenBank accession numbers of *bws opsin*, *uvs opsin* are XP_046438769.1, EFX75461.1. Lpol: *Limulus polyphemus*: the GenBank accession numbers of *ocellar opsin*, *uvs* and *lateral eye opsin* are NP_001301089.1, AEL29244.1, NP_001301044.1. Pcla: *Procambarus clarkia*: the GenBank accession numbers of *opsin* is AAB25036.1. Hsan: *Hemigrapsus sanguineus*: the GenBank accession numbers of *opsin 1* and *opsin 2* are BAA09132.1, BAA09133.1. Hera: *Heliconius erato*: the GenBank accession numbers of *lws opsin*, *bws opsin* and *uvs opsin* are AAY16540.1, AAY16539.1, AAY16537.1. Lser: *Lasioderma serricorne*: the GenBank accession numbers of *lws opsin*, *uvs opsin* are QPF71148.1, QPF71149.1 (Table S3).

Three opsin genes were identified from the *P. astrigera* transcriptome database. Their mRNA sequences are available in NCBI GenBank. The predicted amino acid sequences of these genes contain conserved domains typical of G-protein-coupled receptors, including seven transmembrane domains and protein kinase C phosphorylation sites (Figure 3A). Phylogenetic analysis shows that the visual protein genes of arthropods are clearly classified into three categories: medium long-wave-sensitive opsins, short-wavelength- and UV-sensitive opsins, and non-visual functional opsins. The *PastRH1* and *PastRH2* genes are grouped with medium to long wave sensitive opsins, while the PastRH3 gene is grouped with short-wavelength and UV-sensitive opsins. Among them, the opsin gene of *P. astrigera* is closely related to the opsin gene of *C. salei*. The opsins genes with the same function in the Arachnida are clustered together, and in addition, the RH2 and RH3 genes in Arachnida are clearly clustered together with the visual protein of Limulus polyphemus, which belongs to another class, Merostomata. The clustering of RH1 and RH2 in *P. astrigera* is closely related to the mid to long wave sensitive visual proteins in Insecta, representing the relationship between the Insecta and Arachnida arthropod groups (Figure 3B). The lysine residue in the second transmembrane domain of *PastRH3* is known to confer ultraviolet sensitivity [31]. 

### 3.2. Expression Profiles Across Tissues and Developmental Stages

**Figure 4:**
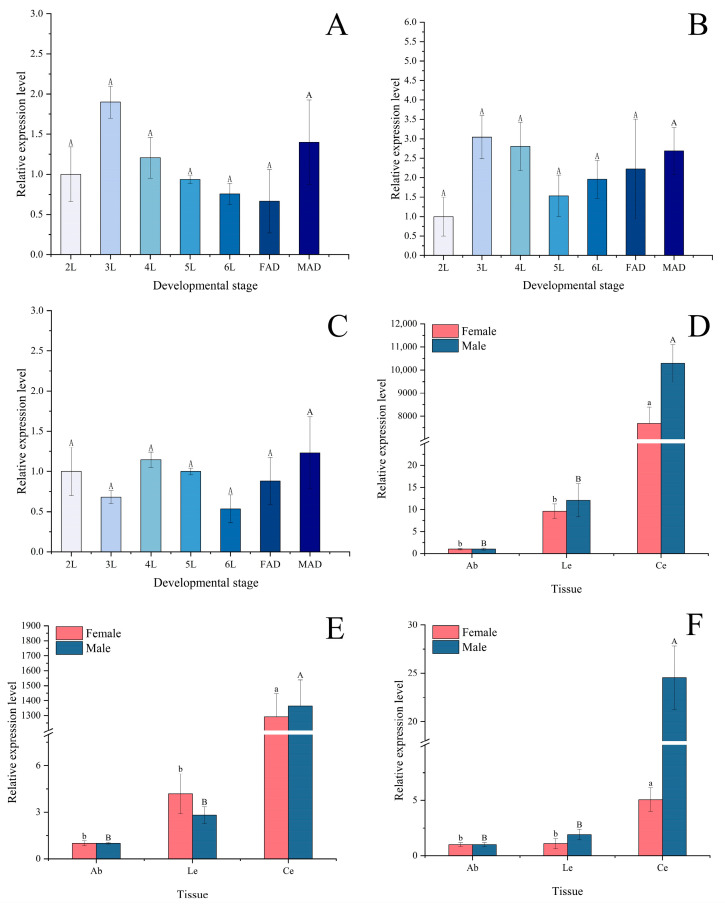
The relative expression levels of *PastRH* at different developmental stages and organizations of *P. astrigera*. (**A**) The relative expression levels of *PastRH1* at different developmental stages. (**B**) The relative expression levels of *PastRH2* at different developmental stages. (**C**) The relative expression levels of *PastRH3* at different developmental stages. (**D**) The relative expression levels of *PastRH1* at different organizations in male and female adult spiders of *P. astrigera*. (**E**) The relative expression levels of *PastRH2* at different organizations in male and female adult spiders of *P. astrigera*. (**F**) The relative expression levels of *PastRH3* at different organizations in male and female adult spiders of *P. astrigera*. FAD: adult female, MAD: adult male, Ab: abdomen, Le: leg, Ce: Cephalothorax, The relative expression level was calculated using the 2^−ΔΔCt^ method. Data are presented as mean ± standard error. (**A**–**C**) were calculated using single factor ANOVA test followed by Tukey’s HSD multiple comparisons, a value of *p* < 0.05 was considered statistically significant. (**D**–**F**) were calculated using single factor ANOVA test followed by Tamhane’s T2 multiple comparisons, a value of *p* < 0.05 was considered statistically significant, compared only within the male and female groups. Capital letters and lowercase letters represent differences within male and female groups.

The three opsin genes of *P. astrigera* were expressed across all developmental stages examined. No significant differences in the expression levels of *PastRH1* (Figure 4A), *PastRH2* (Figure 4B), or *PastRH3* (Figure 4C) were observed among the second to sixth instar spiderlings and adult males and females (*p* > 0.05). However, the expression levels of *PastRH1* and *PastRH2* showed a noticeable increase from the second to the third instar.

In male and female *P. astrigera*, the three opsin genes (*PastRH1* (Figure 4D), *PastRH2* (Figure 4E), and *PastRH3* (Figure 4F)) were expressed in the cephalothorax, abdomen, and legs (Figure 4). Among these tissues, all three genes showed significantly higher expression levels in the cephalothorax compared to the abdomen and legs (*p* < 0.05), while no significant differences were observed between the abdomen and legs (*p* > 0.05).

### 3.3. Expression Induced by Different Light Wavelengths

**Figure 5:**
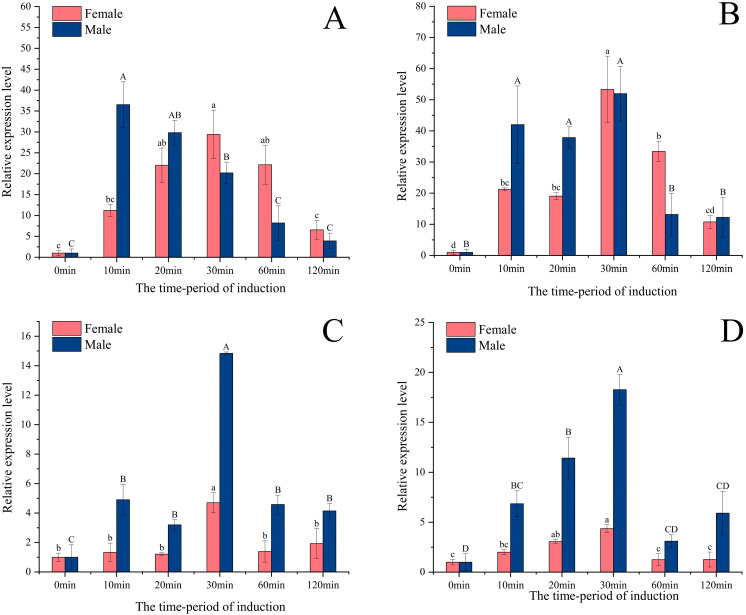
The relative expression of *PastRH* at different wavelengths light of *P. astrigera*. (**A**) The relative expression levels of *PastRH1* at the green light (520–525 nm). (**B**) The relative expression levels of *PastRH2* at the green light (520–525 nm). (**C**) The relative expression levels of *PastRH3* at the blue light (460–465 nm). (**D**) The relative expression levels of *PastRH3* at the ultraviolet light (370–375 nm), The relative expression level was calculated using the 2^−ΔΔCt^ method. Data are presented as mean ± standard error. (**A**–**D**) was calculated using single factor ANOVA test followed by Tukey’s HSD multiple comparisons, a value of *p* < 0.05 was considered statistically significant. Capital letters and lowercase letters represent differences within male and female groups.

Under green light (wavelength 520–525 nm) stimulation, the expression level of the *PastRH1* gene (Figure 5A) in adult male and female *P. astrigera* spiders showed a tendency of increasing first and then decreasing over the treatment period. Specifically, the expression level in females peaked at 30 min, while in males it reached its highest level at 10 min. For the *PastRH2* gene (Figure 5B), expression in females was highest at 30 min, showing a significant difference compared to other time points (*p* < 0.01). In males, however, there was no significant difference in expression levels among the 10, 20, and 30 min treatment groups (*p* > 0.05).

Under blue (460–465 nm; Figure 5C) and ultraviolet (370–375 nm; Figure 5D) light stimulation, the expression of *PastRH3* in both male and female adults exhibited a similar temporal pattern, with levels increasing and then decreasing over time. Expression peaked at 30 min post-treatment and was significantly higher than at other time points (*p* < 0.01).

### 3.4. Functional Validation of Opsin Genes via RNAi

**Figure 6:**
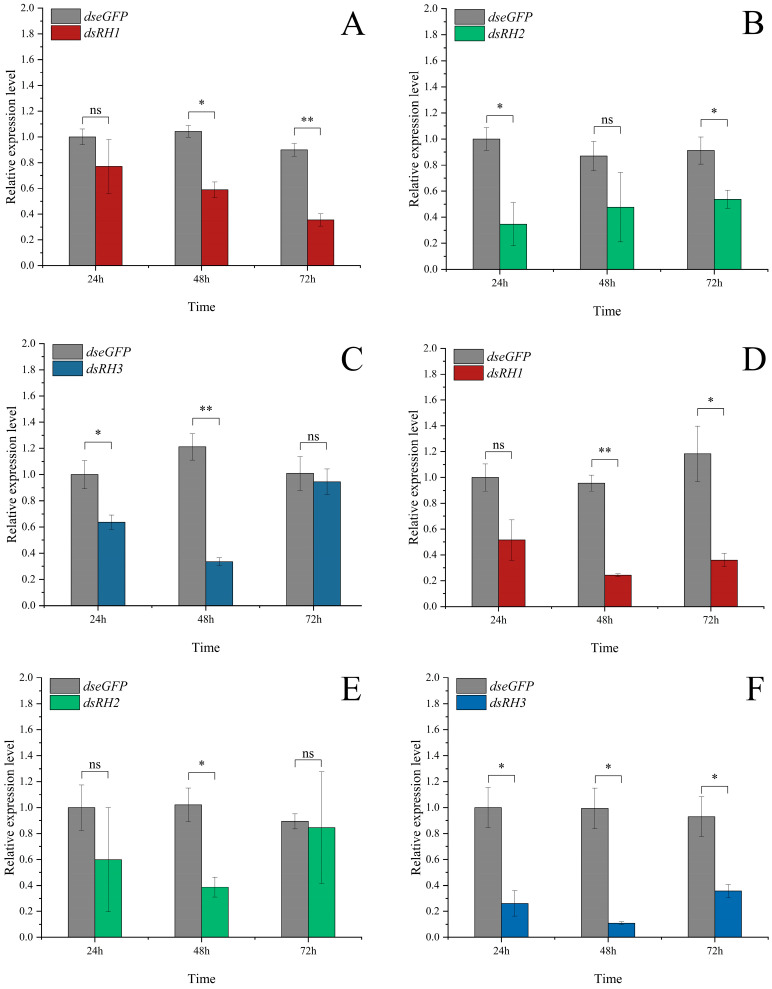
Effect of RNA interference of *PastRH* on the relative expression level of *PastRH* of *P. astrigera* adult females and males. (**A**) The relative expression level of *PastRH1* of adult females. (**B**) The relative expression level of *PastRH2* of adult females. (**C**) The relative expression level of *PastRH3* of adult females. (**D**) The relative expression level of *PastRH1* of adult males. (**E**) The relative expression level of *PastRH2* of adult males. (**F**) The relative expression level of *PastRH3* of adult males. Data are presented as mean ± standard error. Independent sample *t*-test *p*-value, Significant differences are indicated by asterisks (**: *p* < 0.01, *: *p* < 0.05, ns: not significant).

Opsins play a crucial role in *P. astrigera*’s selection of light sources with different wavelengths. To verify the function of opsin genes, dsRNA encapsulated in nanomaterials was applied via droplet administration to interfere with gene expression, using *dseGFP* as a negative control (Table S4). The results showed that the expression level of *PastRH1* decreased by 60.5% in females at 72 h post-treatment (Figure 6A), showing a highly significant difference compared to the control group (*p* < 0.01); in males, expression decreased by 74.6% at 48 h (Figure 6D), also showing a highly significant difference (*p* < 0.01).

For *PastRH2*, expression in females decreased by 65.3% at 24 h (Figure 6B), showing a significant difference compared to the control (*p* < 0.05); in males, expression decreased by 62.1% at 48 h (Figure 6E), showing a significant difference compared to the control (*p* < 0.05).

The expression level of *PastRH3* decreased by 73.1% in females at 48 h (Figure 6C), showing a highly significant difference (*p* < 0.01); in males, expression was reduced by 89.6% at 48 h (Figure 6F), showing a significant difference (*p* < 0.05).

Using red light (625–635 nm), which is invisible to *P. astrigera*, as the control light source, phototaxis preference experiments were conducted under different wavelength light sources. The results showed that, compared to the *dseGFP* control group, the phototactic preference of female *P. astrigera* for green light (520–525 nm) was abrogated after treatment with *dsRH1* and *dsRH2*. After treatment with *dsRH3*, the strong phototactic preference for blue light (460–465 nm) and the highly significant preference for ultraviolet light (370–375 nm) were reduced to non-significant differences.

**Figure 7:**
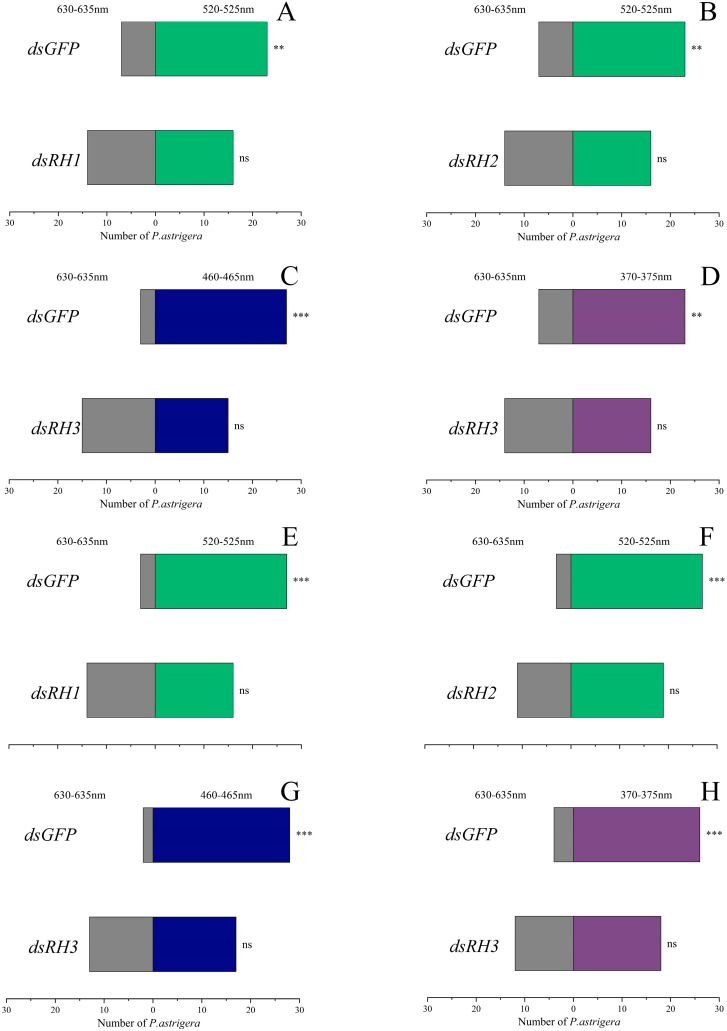
Effect of RNAi of *PastRH* on behavioral selectivity of *P. astrigera*. (**A**) RNAi of *PastRH1* reduces the behavioral selectivity of adult females at the green light (520–525 nm). (**B**) RNAi of *PastRH2* of adult females at the green light (520–525 nm). (**C**) RNAi of *PastRH3* of adult females at the blue light (460–465 nm). (**D**) RNAi of *PastRH3* of adult females at the purple light (370–375 nm). (**E**) RNAi of *PastRH1* of adult males at the green light (520–525 nm). (**F**) RNAi of *PastRH2* of adult males at the green light (520–525 nm). (**G**) RNAi of *PastRH3* of adult males at the blue light (460–465 nm). (**H**) RNAi of *PastRH3* of adult males at the purple light (370–375 nm). The control was red light at 625–635 nm. Significant differences are indicated by Chi-square test (***: *p* < 0.001, **: *p* < 0.01, ns: not significant).

In males, after treatment with *dsRH1* and *dsRH2*, the phototactic preference for green light (520–525 nm) was abolished. After treatment with *dsRH3*, the strong preference for blue light (460–465 nm) and ultraviolet light (370–375 nm) was diminished as well (Figure 7).

## 4. Discussion

As an active hunting spider, wolf spiders possess relatively well-developed vision. They are sensitive to medium-to-long-wavelength green light and short-wavelength ultraviolet (UV) light, but not to long-wavelength red light. In this study, three vision-related genes were identified from the *P. astrigera* transcriptome database: *PastRH1* and *PastRH2*, which are sensitive to medium-to-long-wavelength green light, and *PastRH3*, which is UV-sensitive. These findings differ from those in insects, which typically possess three types of opsins: UV-sensitive, blue-sensitive, and medium-to-long-wavelength-sensitive opsins. Interestingly, phylogenetic analysis revealed that *PastRH3* in *P. astrigera* clustered not only with UV-sensitive opsins but also with blue-sensitive opsins.

Similar findings have been reported in insects. Markus proposed that in aphids and planthoppers, UV-sensitive opsins may have shifted from ancestral UV peaks to derived blue-sensitive ones to compensate for the loss of blue opsins [32]. Kirchner’s research on the green peach aphid (*Myzus persicae*) supported this idea, showing that the species exhibits trichromatic vision despite lacking a dedicated blue opsin [33]. However, the exact molecular mechanism behind this compensation is still unclear, and further research is needed to determine whether *P. astrigera*’s vision has the same compensation mechanism to achieve better color vision.

Most opsin genes are not only expressed in photoreceptors but also in other tissues, and this has been confirmed for the first time in spiders through this study. RT-qPCR analysis revealed that all three opsin genes were expressed in the cephalothorax, abdomen, and legs of *P. astrigera*, with significantly higher expression levels in the cephalothorax compared to the abdomen and legs [34,35,36]. The eight simple eyes of *P. astrigera* are concentrated at the anterior region of the cephalothorax and serve as the primary organs for receiving visual stimuli, playing a key role in visual behavior. However, other parts of the body also have expression of opsin genes, but their specific functions are not yet clear. The reason for the expression of opsin gene in the part without visual function needs to be studied later to determine its specific function. Further studies are needed to clarify their specific functions outside of vision.

The transcription levels of the three opsin genes were upregulated to varying degrees under exposure to specific colored light, with clear sexual dimorphism observed in their expression. The expression levels of *PastRH1*, *PastRH2*, and *PastRH3* all increased following stimulation with different wavelengths of light, indicating that these genes are responsive to external environmental cues. Similar findings have been reported in other studies, where the expression of opsin genes in *Mythimna separata*, *Tribolium castaneum*, and *Spodoptera frugiperda* was influenced by light color or intensity [37,38,39]. Organisms exposed to increased light levels tend to exhibit enhanced opsin gene expression—a phenomenon also observed in insects such as *Ceratosolen solmsi*, *Apis mellifera*, *Helicoverpa armigera*, and *Acyrthosiphon pisum* [40,41,42,43].

However, there are also differences in the expression levels of the opsin gene between males and females in *P. astrigera*. Field and laboratory observations have shown that males locate prey and handle food more quickly than females, and display greater agility during courtship, mating, and predator avoidance behaviors, with a wider range of activity, females optimize their feeding efficiency by extending visual assessment [44]. The relative expression levels of female and male visual proteins have different response times to light stimuli. Under different light wavelengths, the expression of green-sensitive *PastRH1* and *PastRH2* genes peaked at 10 min post-treatment in males, while in females, peak expression occurred at 30 min, further supporting the presence of sex-specific differences in visual gene responsiveness.

All three opsin genes were expressed across different developmental stages of *P. astrigera*, although the differences in expression levels were not statistically significant. However, the expression levels of *PastRH1* and *PastRH2* in third-instar spiderlings were notably higher than those in second-instar individuals. This may be attributed to behavioral differences between the stages: second-instar spiderlings spend most of their time under maternal care, whereas third-instar spiderlings lead an independent life. At this stage, they must locate food, find shelter, and avoid predators on their own, which likely requires enhanced visual capabilities, thereby resulting in elevated opsin gene expression.

The mechanisms underlying phototactic behavior in arthropods are complex, with opsins playing a central role in light perception. In this study, both male and female *P. astrigera* adults exhibited significant phototactic responses to ultraviolet, blue, and green light. However, after RNAi-mediated knockdown of the three opsin genes, these significant preferences disappeared, indicating that opsins are critically involved in the regulation of phototactic behavior as well as maternal care behaviors such as brood carrying and guarding. In integrated pest management (IPM), many control strategies exploit the phototaxis of insect pests, but these methods often result in unintended harm to natural enemies. The suppression of phototactic behavior in spiders via opsin gene interference offers a potential approach to reduce such non-target impacts and provides new insights for the development of more selective and ecologically sound pest control strategies.

## 5. Conclusions

In *P. astrigera*, vision plays a crucial role in various behavioral processes, including predation, courtship and mating, maternal care, development, predator avoidance, and the search for suitable habitats. The expression levels of opsin genes are key to supporting these visually guided functions. Our results demonstrate that *P. astrigera* exhibits trichromatic peak sensitivity but lacks a distinct blue-sensitive opsin gene.

Our research reveals the phenomenon that three opsin genes showed varying degrees of transcriptional upregulation in response to specific wavelengths of light, with notable sexual dimorphism in expression patterns.

This study provides valuable insights into the visual basis of maternal care behaviors in spiders. However, the function of opsin gene expression in non-visual tissues such as the abdomen and legs remains unclear and warrants further investigation.

In addition, UV-sensitive opsin has blue light sensitivity in behavioral verification. Whether UV-sensitive opsin compensates for the loss of blue-sensitive opsin also needs to be studied.

## Data Availability

The raw data supporting the conclusions of this article will be made available by the authors on request.

## Supplementary Materials

The following supporting information can be downloaded at: https://www.mdpi.com/article/10.3390/insects16060595/s1, Figure S1: Electrophoretogram of *PastRHs* cloning from *P. astrigera*; Table S1: The sequence information of *PastRHs*. Table S2: Primers for fatty acid synthase of *P. astrigera* in this study; Table S3: Species and GenBank accession numbers of Opsin Genes used in the phylogenetic analysis in this study. Table S4: The exact knockdown efficiency (percentage reduction) for opsin gene. Video S1: Spider choice behavior video.
