# Supplementary material for: Functional Study of Opsin Genes in Pardosa astrigera (Araneae: Lycosidae)
Source: Insects. 2025 Jun 5;16(6):595. doi: 10.3390/insects16060595 (PMC12193414; doi:10.3390/insects16060595)

Table S1. The sequence information of *PastRHs*.

PastRH1 Nucleotide	TTCCAGGGTCGGATAAGGCGCTCAAGAGATATAAGAACTTAATTTTGATC<br>TGATATGTCAGTCTTCGATAATCACATGAAACAAAAACGATGAATAATGA<br>CATCGATTAAGACTCCACAAAAACAATCTCCAAATTTCGGACTGCCTCAA<br>ACGCTGTGAACCCAACACACCGGCAACATGATTAAACCAATGATGATGCC<br>TCACGCCGAGAAGTACACCTTCTCCGAGATGTTCTCCGATCCCTACGAGA<br>AGAACGCGTCCATCGTGGACCTTCTCCCGGAGAACATGCTCTACATGATC<br>CACGAGCACTGGTACGGCTTCCCTCCCATGAAGGCGGCGTGGCACTACA<br>TCTTGGGTGTGACAATCATGATACTGGGGATCATCAGCGTCAGCGGCAAC<br>GGCATCGTCCTCTACCTCATGACCAGCTGCAAGGGACTGCGCTCCCCCAA<br>CAACCTGCTCGTCATGAACCTCGCCTTCTCGGACTTCTGCATGATGGCCT<br>TCATGATGCCCACCATGTCGGCCAACTGCTTCGGCGAGACGTGGATCCTG<br>GGGCCGCTCATGTGCGAGATCTACGGCATGTTCGGATCCCTCTTCGGTTG<br>CGGATCCATCTGGAGCATGGTCTTTATCACCCTAGACCGCTACAACGTCAT<br>CGTGCGGGGAATGGCCGCTAAACCACTCACCAAGTCCAGGGCCCTAGTC<br>GCCATCCTTTTCATATGGATATATGCCGTAGGATGGACCATCGCACCTATGT<br>ACGGATGGAGCAGATATGTACCCGAGGGCGCAATGACGGGTTGCACGGT<br>CGACTACGTGAGCTCTAACGCCAACAACATGTCTTACCTGGTGGTTTACG<br>CGATCTTTGTGTACTTCGTGCCCCTGTGCACCATGATCTACTGCTACACGT<br>TCATCGTCATGCAGGTGGCTTCGCACGAGAAACAGCTGAGGGAACAGGC<br>GAAGAAGATGAACATCGCTTCGCTCAGAGCCAACGCAGACAATCAGAAG<br>ACCAGCGCAGAGTTCAGGCTCGCTAAGGTGGCCTTCACGACGGTGATTC<br>TGTGGTTCATGGCGTGGACGCCCTACCTGATCCTATCGCTCCTCGGCATTT<br>TCACCGACCGCTCCCAAATCACCCCGATGTCGACGATCTGGGGTGCGGT<br>GTTCGCGAAGGCGAGCGCCTGCTACAATCCCATCGTGTACGGCATCAGCC<br>ATCCCAAGTACCGGGCCGCGCTCAACGAGAAGTTCAAGTGCCTCGGCGC<br>GCCCGAGGCGCCCTCCAAAGGAGATGCCGCCTCCACGGTCTGCAGCGAG<br>ATGGACAAGAGTGTCGGAGAATAAGACAATACTCTTATCGCAACCTGTAA
PastRH1 Protein	MIKPMMMPHAEKYTFSEMFSDPYEKNASIVDLLPENMLYMIHEHWYGFPP<br>MKAAWHYILGVTIMILGIISVSGNGIVLYLMTSCKGLRSPNNLLVMNLAFSD<br>FCMMAFMMPTMSANCFGETWILGPLMCEIYGMFGSLFGCGSIWSMVFITL<br>DRYNVIVRGMAAKPLTKSRALVAILFIWIYAVGWTIAPMYGWSRYVPEGAM<br>TGCTVDYVSSNANNMSYLVVYAIFVYFVPLCTMIYCYTFIVMQVASHEKQL<br>REQAKKMNIASLRANADNQKTSAEFRLAKVAFTTVILWFMAWTPYLILSLL<br>GIFTDRSQITPMSTIWGAVFAKASACYNPIVYGISHPKYRAALNEKFKCLGA<br>PEAPSKGDAASTVCSEMDKSVGE
PastRH2 Nucleotide	GCGGTTCCTTGTGGTTCTGTTGGCTAGCATCCTCTGCAGGAGATTCACAG<br>CGCTCCTTTGCGGGTGTCTCTTGTAGATAAAGATTTCCGAGTTTGAGCTTT<br>GATTTACCTTCTTCAGTCATGTCTTCCCAGGCCTTTAATAACGCCTTCATG<br>ATCCCTCGGGGATATCAACCACCACCAGTCAATCTTGGCCGTCCAGTGCC<br>TTATAACGGTGCCTTCTACCCTTACCAATCAAATGCTACCGTTGTGGACAC<br>CGTGCCTGCTGAGATCTTGCACATGGTCCACGAACACTGGTACCAATTTC<br>CACCAATGAATCCCTTGTGGCACAGCCTGCTCGGGGTTGCCATGATTGTG<br>TTGGGAATCATCAGTGTTATCGGCAATGGAATGGTTGTCTACCTCATGTCT

	ACCACCAGGAGCCTCAAGACACCGACTAACATGTTGATCGTAAACTTGG<br>CAtTCTCGGATTTCTGCATGATGGCCTTCATGATGCCCACTATGGCCGCTA<br>ATTGTTTCGCCGAAACATGGATCTTGGGACCGTTCATGTGTGAAGTTTATG<br>GAATGGTTGGCAGTCTTTTTGGCTGCGTTTCCATCTGGACCATGGTGATG<br>ATCGCATTCGACAGATACAACGTCATCGTTAATGGTATGTCCGCAGAACCT<br>CTGACAAGCAAGAAGGCGGCACTTCAGATCTTCCTCGTCTGGGCCTGGT<br>CTGCCGTCTGGACCCTTCTCCCCTTTTTCGGATGGAACAGGTATGTACCT<br>GAGGGCAACATGACCAGCTGCACCATCGATTATCTCACCAAAGACCCTGC<br>TTCATCTTCCTACGTCATCATGTATGGTGTTGCCGTATACTTTGCTCCTTTG<br>GCCACTCTGATCTATAACTACACCTTCATTGTCAAATCTGTTGCCACCCAC<br>GAGCAGCAGCTGAGAGAGCAAGCCAAGAAGATGAACGTTTCTTCCCTCC<br>GAGCTAATGCTGATCAACAAAAGCAGTCAGCAGAATGcCGTCTTGCTAAG<br>GTCGCAATGATGACCGTTGGCTTATGGTTCATCGCATGGACACCCTACCTC<br>AGCATCGCCTGGAGTGGAATTTTCTCCTCAAGAAAACATCTTACTCCATT<br>GGCCACCATCTGGGGAGCTGTCTTCGCCAAAGCGGTCGCCGTGTACAAT<br>CCTATTGTCTACGGTATCAGCCATCCCAAATACAGAGCGGCTCTTCACCA<br>GAGATTCCCGAGTCTAGCTTGCGCTTCAGATAATGGTAACCATGGAAACG<br>ACAACAGGTCCGAAGCGACCGTCGTTATGGACGAAAAGCCACCAAAGA<br>ACCCTGAAGCTTAAATTAAAAAAAAAATTATGTCTTTTTGTTTAAGCTTGT<br>AAAGTTTACGCGCTTTTAAAGATATATTTTTCACGATTATTGGTTTCGATGC<br>TTCGCACTAGA
PastRH2<br>Protein	MSSQAFNNAFMIPRGYQPPPVNLGRPVPYNGAFYPYQSNATVVDTVPAEIL<br>HMVHEHWYQFPPMNPLWHSLLGVAMIVLGIISVIGNGMVVYLMSTTRSLK<br>TPTNMLIVNLAFSDFCMMAFMMPTMAANCFAETWILGPFMCEVYGMVGS<br>LFGCVSIWTMVMIAFDRYNVIVNGMSAEPLTSKKAALQIFLVWAWSAVWTL<br>LPFFGWNRYVPEGNMTSCTIDYLTKDPASSSYVIMYGVAVYFAPLATLIYNY<br>TFIVKSVATHEQQLREQAKKMNVSSLRANADQQKQSAECRLAKVAMMTV<br>GLWFIAWTPYLSIAWSGIFSSRKHLTPLATIWGAVFAKAVAVYNPIVYGISHPK<br>YRAALHQRFPSLACASDNGNHGNDNRSEATVVMDEKPPKNPEA
PastRH3<br>Nucleotide	GCTGGCAGATGTTGAACTCTTCATCTCACCCAGCACTTCTGGACGACATA<br>AGTCCTCCATCATGGTGCTACGAGACGCGTTTCAATGGCTGGAACACCCC<br>GCCCGATGTCTACGTGAGCCCTTACTGGAGGCAGTTCCGGGCGCCAGCG<br>CCCTACTTGCACTATCTGCTTGGCATTCTCTACATTGCCCTAACGATTATCT<br>CATGTGTCGGGAACGGAATGGTCATGTATATCTTCATGGTAGCCAAAAGC<br>TTGAGGACACCAGCAAATATGTTTGTAATCGGACTTGCAATGTCAGATCT<br>ACTTATGATGGCTAAAACACCAGTTTTCATTTACAATTGTTTCCAGCTTGG<br>GCCTGTTTTTGGAAATTTAGGTTGCACACTTTACGGAGTTGTAGGTGCGT<br>ATTCAGGCCTCGGATCTGCCTTCTGCAATGCCATCATCGCCTACGATCGAT<br>TTAGAGTCATAGTGCATCCCTTCAGCAAGTCAGGAATGTCTATGACCAAA<br>GCCATCATCATGCTGGTTCTCATTTACGTCTACATAACACCGTTTGCTCTG<br>CTCCCGGCTTTCCACATTTGGAGTCGGTTCGTGCCGGAAGGGTTTCTCAC<br>CAGTTGCGCGGCCGACTTCTTCATGCACGACTTCAACGGCAGGTCATACA<br>TCGTGGGCACCTGGTTCTTCGGCTGGTTCATCCCTATCAGCATAGTGCTGT<br>TCTGCTACGCGCGGATATTCATGGCCGTGAGGAATCACGAAAACCAAATC

	AAGGAACAGGCTCGCAAAATGAACGTGGACAGCATCCGCTCCAATGCAG<br>CCGTGAAGAGTTCGTCGGCCGAGGTACGAATCGCCAAGACGGCCTTCTG<br>CGTGATCGTCCTTTTCCTCTTCTCTTGGGTCCCTTACATCTCTGTGGCCTT<br>CATTGCTGGATTCTCGGATCCCAAGACTCGCCGGATTACGCCCGTGCTGT<br>CAATGATACCAGCTCTCACCCTGAAGGCGTCGGCCTGCTTCGATCCATTC<br>TTCTACGCCATCAGCCACCCGAGATACCGACAGGAGCTGCAGAACCGGG<br>TTCCCTGGCTCTGCATCAACGAGAAGGCAGAGGCGGCACACGCTAACGG<br>AACCTGCGACGAAGTCTCCAAGACCACGGAACACCCCTGAGGACGGAG<br>GGGGAAACAAGGCACTTTTGTTAACCACGCTTCGCAATGATAG
PastRH3 Protein	MLNSSSHPALLDDISPPSWCYETRFNGWNTPPDVYVSPYWRQFRAPAPYLH<br>YLLGILYIALTIISCVGNGMVMYIFMVAKSLRTPANMFVIGLAMSDLLMMAK<br>TPVFIYNCFQLGPVFGNLGCTLYGVVGAYSGLGSAFCNAIIAYDRFRVIVHPF<br>SKSGMSMTKAIIMLVLIYVYITPFALLPAFHIWSRFVPEGFLTSCAADFFMHD<br>FNGRSYIVGTWFFGWFIPISIVLFCYARIFMAVRNHENQIKEQARKMNVDSIR<br>SNAAVKSSSAEVRIAKTAFCVIVLFLFSWVPYISVAFIAGFSDPKTRRITPVLS<br>MIPALTLKASACFDPFFYAISHPRYRQELQNRVPWLCINEKAEAAHANGTCD<br>EVSKTTEHP

Table S2. Primers for fatty acid synthase of *P. astrigera* in this study

	Primer sequences (5'-3')	Primer usage	Product length
PastRH1-F	TTCCAGGGTCGGATAAGG	*PastRHs* cloning (Full length)	1467bp
PastRH1-R	TTACAGGTTGCGATAAGAGTA		
PastRH2-F	GCGGTTCCTTGTGGTTCTGTT		1443bp
PastRH2-R	TCTAGTGCGAAGCATCGAAACC		
PastRH3-F	GCTGGCAGATGTTGAACTCTT		1297bp
PastRH3-R	CTATCATTGCGAAGCGTGGTT		
q-*PastRH1*-F	AACAGGCGAAGAAGATGA	RT-qPCR	146bp
q-*PastRH1*-R	GAGCGATAGGATCAGGTAG		
q-*PastRH2*-F	TCAAGACACCGACTAACAT		152bp
q-*PastRH2*-R	ACTGCCAACCATTCCATA		

q-*PastRH3*-F	AGTTGTAGGTGCGTATTCA		108bp
q-*PastRH3*-R	ATAGACATTCCTGACTTGCT		
β-actin-F	GCAATCCTTCGTTTGGACTT		102bp
β-actin-R	TTCTCTTTCAGCAGTGGTAGTGA		
ds*PastRH1*-F	TAATACGACTCACTATAGGGCCAT CTGGAGCATGGTCTTT	dsRNA	341bp
ds*PastRH1*-R	TAATACGACTCACTATAGGGTTCC CTCAGCTGTTTCTCGT		
ds*PastRH2*-F	TAATACGACTCACTATAGGGAGTC AGCAGAATGCCGTCTT		329bp
ds*PastRH2*-R	TAATACGACTCACTATAGGGAGGG TTCTTTGGTGGCTTTT		
ds*PastRH3*-F	TAATACGACTCACTATAGGGCTTC AACGGCAGGTCATACA		410bp
ds*PastRH3*-R	TAATACGACTCACTATAGGGGCAG CTCCTGTCGGTATCTC		
ds*GF*P-F	TAATACGACTCACTATAGGGGTGT TCAATGCTTTGCGAGA		423bp
ds*GFP*-R	TAATACGACTCACTATAGGGAAAG GGCAGATTGTGTGGAC		

Note: The T7 promoter sequences were underlined.

Table S3. Species and GenBank accession numbers of Opsin Genes used in the phylogenetic analysis in this study.

Species	GenBank Accession Number
Cupiennius salei rhodopsin 1	CCO61973.1
Cupiennius salei rhodopsin 2	CCO61974.1
Cupiennius salei rhodopsin 3	CCO61975.1
Drosophila melanogaster NINAE	NP_524407.1
Drosophila melanogaster rhodopsin 2	NP_524398.1
Drosophila melanogaster rhodopsin 3	NP_524411.1
Drosophila melanogaster rhodopsin 4	NP_476701.1
Drosophila melanogaster rhodopsin 5	NP_477096.1
Drosophila melanogaster rhodopsin 6	NP_524368.5
Drosophila melanogaster rhodopsin 7	NP_524035.2
Apis mellifera LWS	NP_001011639.2
Apis mellifera BWS	AAC13417.1
Apis mellifera UVS	AAC47455.1
Hasarius adansoni kumopsin 1	BAG14330.1
Hasarius adansoni kumopsin 2	BAG14331.1
Hasarius adansoni kumopsin 3	BAG14332.1
Plexippus paykulli kumopsin 1	BAG14333.1
Plexippus paykulli kumopsin 2	BAG14334.1
Plexippus paykulli kumopsin 3	BAG14335.1
Pardosa astrigera rhodopsin 1	PV524665.1
Pardosa astrigera rhodopsin 2	PV524666.1
Pardosa astrigera rhodopsin 3	PV524667.1
Daphnia pulex BWS	XP_046438769.1

Daphnia pulex UVS	EFX75461.1
Heliconius erato BWS	AAY16539.1
Heliconius erato UVS	AAY16537.1
Heliconius erato LWS	AAY16540.1
Limulus polyphemus ocellar opsin	NP_001301089.1
Limulus polyphemus UVS	AEL29244.1
Limulus polyphemus lateral eye opsin	NP_001301044.1
Procambarus clarkia opsin	AAB25036.1
Hemigrapsus sanguineus opsin 1	BAA09132.1
Hemigrapsus sanguineus opsin 2	BAA09133.1
Lasioderma serricorne LWS	QPF71148.1
Lasioderma serricorne UVS	QPF71149.1

Table S4. The exact knockdown efficiency (percentage reduction) for opsin gene.

Genes	Gender	Time	The exact knockdown efficiency
PastRH1	Female	24h	22.97%
		48h	43.57%
		72h	60.52%
	Male	24h	48.48%
		48h	74.59%
		72h	69.53%
PastRH2	Female	24h	65.33%
		48h	45.18%
		72h	41.05%
	Male	24h	40.15%

		48h	62.11%
		72h	5.43%
PastRH3	Female	24h	36.43%
		48h	72.72%
		72h	6.30%
	Male	24h	73.91%
		48h	89.06%
		72h	57.73%

Figure S1. Electrophoretogram of *PastRHs* cloning from *P. astrigera*

Note: Lane M is the 2KB Marker, lane 1 is *PastRH1*， lane2 is *PastRH2* and lane is *PastRH3*.

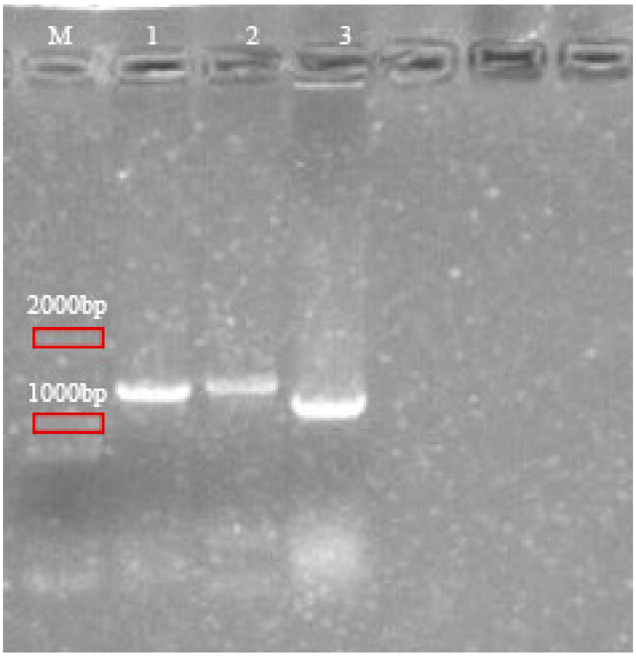

Supplement: Supplementary file 1 [file insects-16-00595-s001.zip › supplement.pdf]
